# Cement Mortar Porosity by Modified Analysis of Differential Scanning Calorimetry Records

**DOI:** 10.3390/ma13051080

**Published:** 2020-02-28

**Authors:** Piotr Stępień, Zbigniew Rusin, Karol Skowera

**Affiliations:** Faculty of Civil Engineering and Architecture, Kielce University of Technology, Al. Tysiąclecia Państwa Polskiego 7, 25-314 Kielce, Poland; zbigniew.rusin@tu.kielce.pl (Z.R.); kskowera@tu.kielce.pl (K.S.)

**Keywords:** pore size distribution, differential scanning calorimetry, phase changes, thermoporometry

## Abstract

A modified method of interpreting a heat flux differential scanning calorimetry records in pore structure determination is presented. The method consists of determining the true phase transition energy distribution due to the melting of water during a differential scanning calorimetry (DSC) heating run. A set of original apparatus functions was developed to approximate the recorded calorimetric signals to the actual processes of the water phase transition at a given temperature. The validity of the proposed calorimetric curves-based algorithm was demonstrated through tests on a cement mortar sample. The correct analysis required taking into account both the thermal inertia of the calorimeter and the thermal effects that are associated with water transitions over the fairly narrow temperature ranges close to 0 °C. When evaluating energy distribution without taking the shifts of the proposed modified algorithm into account, the volume of the pores with radii bigger than 20 nm was greatly overestimated, while that of the smaller pores (r_p_ < 20 nm) was underestimated, in some cases by approximately 70%.

## 1. Introduction

Water phase transition in the mortar pore space has long been in the scope of interest and has been investigated by using numerous methods. Differential scanning calorimetry (DSC) seems to be a particularly well-suited technique for this purpose. Thermoporometry through the use of the DSC has been widely applied to study the influence of curing conditions [1,2,3], admixtures [4], additives [5] and the types of cement [6] that are used on the freezing process in cement-based materials. Thermograms and the latent heat of fusion provide information for calculating the content of ice at specific temperatures, the volume of ice in pores, and pore size distribution (only those filled with water). By investigating the amount of heat that is released/supplied to the system at a given temperature, it is possible to determine the pore size distribution, especially over the range between 2 and 50 nm. The theoretical principles of thermoporometry were thoroughly described by Brun et al. [7].

Between the liquid and solid phases in the pore space, there is a curved interphase. The curvature of the meniscus reduces the free energy of the liquid phase and consequently lowers the temperature of the phase transition. The relationship between the phase transition temperature and the radius of the pore with the trapped liquid is described by Equation (1):

(1) $$r_{K}=-\frac{2\cdot M\cdot \sigma_{l-c}\cdot cos\theta }{\Delta H_{fus}\cdot \rho_{l}\cdot ln\left(\frac{T}{T_{0}}\right)}$$

where *r_K_* is the curvature of the solid–liquid interface; *T*_0_ is the freezing temperature of the bulk liquid; *K*, *T* is the pore liquid phase transition temperature; *K*, *M* is the molar mass of the liquid, kg/mol; *ΔH_fus_* is the heat of fusion, J/mol; and *ρ*_l_ is the density of the liquid, kg/m^3^.

The known values of surface tension and absolute free entropy allow for the obtainment of the relation between the pore radius and the phase transition temperature depression. The task of determining such a relation on the basis of available data for particular physical quantities was undertaken by Ishikiriyama [8,9,10], Brun et al. [7], Fagerlund [11], and Landry [12].

Thermoporometry, similarly to other pore space research methods, has its limitations. The presence of curved gas–ice and gas–liquid interphases affects the recorded thermograms [13,14] and their interpretation. The time and manner of soaking, as well as the occurrence of hydrophobic groups in investigated materials, may affect the degree of pore filling [15]. In thermoporometry, the pore space needs to be fully saturated for only a water–ice interface to occur. The most common liquid that is used in this method is distilled water. Compared to other liquids, e.g., benzene [16], it has a relatively high heat of fusion that positively influences the relation of the recorded signal to the "apparatus noise." It may be impossible to amplify the recorded signal with respect to the baseline. This problem can be solved with the use a derivative scanning calorimetry (DeSC). Derivative scanning calorimeters allow for direct access to minor thermal events that are hidden in the signal-to noise-ratio of classical DSCs [17] due to the special design of pans and the generation of the temperature difference between the cells.

At a fixed scanning rate of calorimetric tests, the signals recorded lag behind the actual temperatures at which the processes take place. The discrepancy between the temperature inside the sample and the temperature sensor increases with an increase in the scanning rate [12,18]. When thermoporometry based on these signals is used to calculate the pore radius distribution for ice melting within the pore space, and the delays due to measuring system thermal inertia can significantly affect the results.

The influence of inertia can be reduced by lowering the cooling or heating rate. However, even at a small scanning rate, there is always a certain temperature shift of the recorded signals in relation to the temperatures at which the exo- or endothermic processes occur. Another approach to eliminate the influence of thermal inertia on the temperature of recorded effects is the quasi-isothermal mode (QI-MDSC) in differential scanning calorimetry [19], which allows for the assignment of the obtained results to temperatures. This method is able to handle samples of the order of milligrams in which temperature gradients are small. It is impossible to prepare small representative samples for the examination of macroscopically heterogeneous materials such as cement mortars, concrete, and rock by QI-MDSC. In the case of measuring instruments in which there is a significant difference between the temperature of the external casing (which is responsible for maintaining the programmed temperature) and the central part of the calorimeter in which the samples (such as in the tests described in this paper) are placed, the use of this method would require a considerable extension of the time of the experiment.

An important element that is necessary to make corrections that are related to the thermal inertia of the measuring system when using constant heating or cooling rates is the knowledge of the so-called apparatus function *a*(*E_i,heat_*). In calorimetric tests, the apparatus function allows for the determination of the value of the recorded signal over designated temperature ranges, when the signal is triggered by a single thermal pulse, which is understood as the amount of energy that is released or absorbed by the sample within a very narrow temperature range. Kozłowski [20,21] discussed the application of apparatus functions in the study of the phase transition of water in water–clay systems. In his works, a single apparatus function was used, regardless of the energy pulse value. The total energy of phase transition was broken down into equal components, and appropriate combinations of these components were analyzed, in particular temperature ranges. The result of the calculations was the energy distribution for which the difference between the experimentally determined curve and the curve that was obtained from the convolution of energy distribution with the apparatus function was as small as possible. The idea of Kozłowski’s solution was used to construct modified algorithm. In studies of calorimetric phase transitions of water confined in the pore space of materials such as concrete, mortar, or paste, the effects to thermal inertia of the measuring system have been omitted. Hence, the proposal of the algorithm based on consecutive approximations in which a separate apparatus function is determined for each thermal pulse.

## 2. Apparatus Function

**Figure 1:**
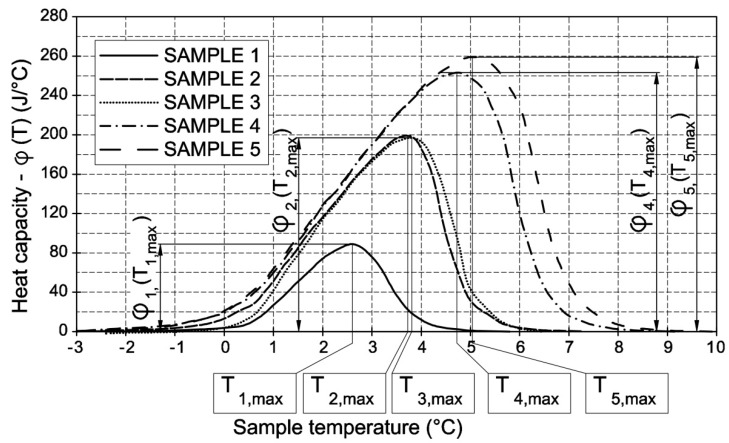
Plots of thermal capacity difference among differential scanning calorimetry (DSC) measurement targets for five samples.

The experimental determination of calorimetric curves is possible through the use of a material in which thermal effects that result from water melting inside the pore space occur within a very narrow temperature range. When this condition is met, the recorded signal is assumed to originate from a single thermal pulse. Because of the spontaneous, random nature of the initial stage of water freezing in the material and the temperature at which the process begins, the signals that are recorded during the ice melting can be used to analyze the volume and dimensions of the pores. In addition, it is known that there may be pore groups with dimensions below 50 nm in which water does not freeze according to the Gibbs–Thomson rule, though spontaneous nucleation is already complete [22]. Only by examining the melting process can the size of the ice-filled pores be correctly assessed. Here, the apparatus functions were experimentally determined for five porous ceramic materials in which more than 97% of the pore volume was made up of pores with dimensions above 0.1 µm (using mercury intrusion porosimetry MIP). Figure 1 shows the values of the recorded thermal effects. The tests were carried out on cylindrical samples (ø13.5 × 70 mm), and which were vacuum saturated with water. The scanning rate was 0.09 C/min. Ice in the pores of the samples melted in a relatively narrow temperature range close to zero degrees Celsius. Therefore, it was possible to assume that the observed heat fluxes were the result of transition processes, which can be referred to as single thermal pulses. In the presented analyses, the temperature range corresponding to the hypothetical “single thermal pulse” was assumed to be 0.05 °C.

The curves of the thermal capacity *φ*(*T*) (Figure 1) were divided into 0.05 °C-wide segments, and the values were determined in the center of each segment/interval *φ_i_*. The *φ*(*T*) curves provided apparatus functions according to the following equation:

(2) $$a_{i}\left(E\right)=\frac{\varphi_{i}}{\sum_{j=1}^{n}\varphi_{j}}$$

where *a_i_* is the value of apparatus function in the *i*-th interval,

(3) $$\overset{n}{\underset{i=1}{\sum }}a_{i}\left(E\right)=1$$

In this way, five apparatus functions were obtained from the φ(T) curves for SAMPLES 1–5, corresponding to their total phase transition energies E_sum_ (Table 1).

**Figure 2:**
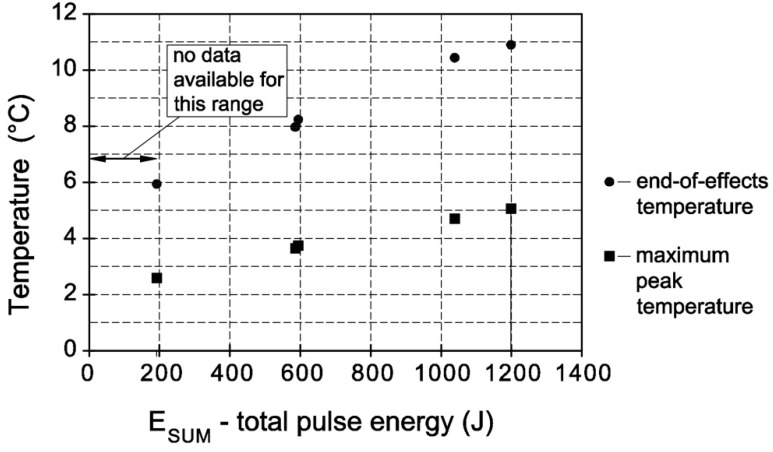
Relationship between the peak maximum and end-of-effects temperature against the total energy of a single thermal pulse.

For the calculations presented for cement mortar, the basic issue is the knowledge of apparatus functions that are generated by single thermal pulses with a total energy amount of less than 193.8 J (Figure 2).

In order to obtain any apparatus function (corresponding to the thermal pulse of *E_sum_* of less than 193.8 J) the curve of thermal capacity *φ*_1_(*T*) for SAMPLE 1 (Figure 1) was transformed according to the equation below.

(4) $$\varphi \left(T\right)=A\varphi_{1}\left(BT\right)$$

where *A* is the variable responsible for the vertical translation of apparatus function, *B* is the variable that is responsible for the horizontal translation of apparatus function, and *T* is temperature.

With the known plot *φ*(*T*) for the given pulse energy *E_sum_*, it is possible to determine the apparatus function in accordance with Equation (1).

**Figure 3:**
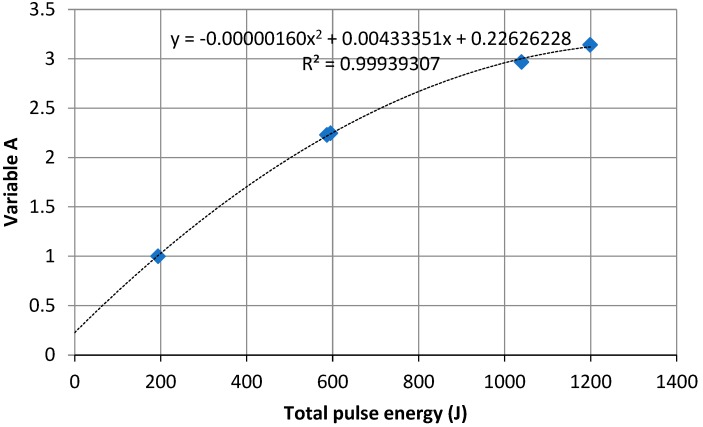
Variable *A* variation against total thermal pulse energy.

Variable A is the ratio of the maximum value on the *φ*(*T*) plot that is triggered by a given heat pulse to the maximum signal value *φ*_1_(*T*_1,max_) for SAMPLE 1 (Figure 1). To determine the Variable A equation, the values of Variable A were determined for ceramic SAMPLES 1–5, and the quadratic trendline was produced (Figure 3).

**Figure 4:**
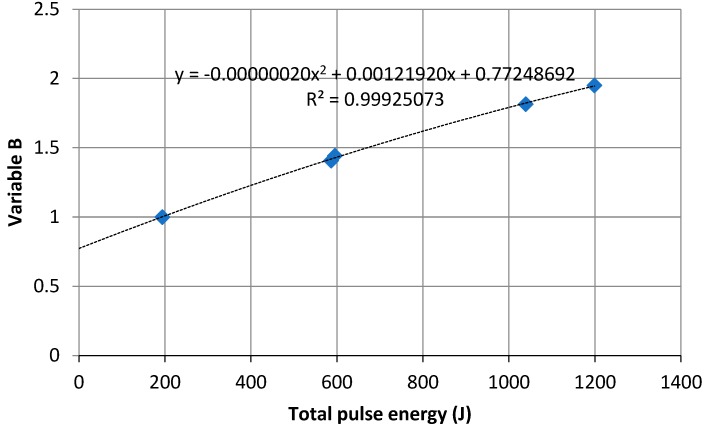
Variable *B* variation against total thermal pulse energy.

Variable B value is the ratio of the maximum peak temperature on the *φ*(*T*) graph, triggered by a given thermal pulse, to the maximum peak temperature *T*_1max_ of SAMPLE 1 (Figure 1). The procedure was the same as for Variable A; the values of Variable B were determined for ceramic SAMPLES 1–5 (points B_1_–B_5_ in Figure 4), and the quadratic trend line was produced for them (Figure 4).

The quadratic equations were used for the *A* and *B* trendline formulas. The authors of this paper are aware that for the extrapolated energy area from 0 to 193.8 J, there may be another relation for Variables *A* and *B*. Nevertheless, at that stage of analysis, the formulas above were used in further calculations.

**Figure 5:**
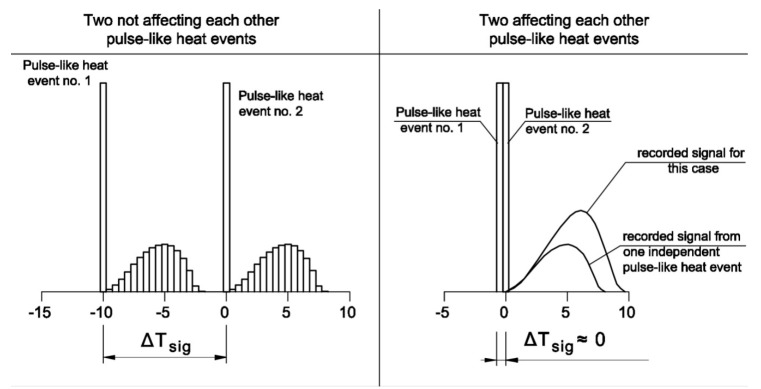
Signals recorded for thermal pulses far away from each other and when the pulses nearly overlap.

The selection of the apparatus function depends not only on the amount of energy of the given thermal impulse for which the function is defined but also on the values of neighboring thermal impulses and the "distance" between them. Whether the thermal impulses influence each other depends on their position with respect to each other on along the temperature axis, which is expressed as ΔT_sig_. In the case of very close location of two peaks (ΔT_sig_ ≈ 0), the recorded effect is the same as in the case of a single peak whose apparatus function is selected on the basis of their cumulative energy (Figure 5). Thus, when determining the apparatus function of a given thermal pulse, one should take into account not only the amount of energy of this peak but also a certain percentage of the value of neighboring peaks, where this value depends on ΔT_sig_.

**Figure 6:**
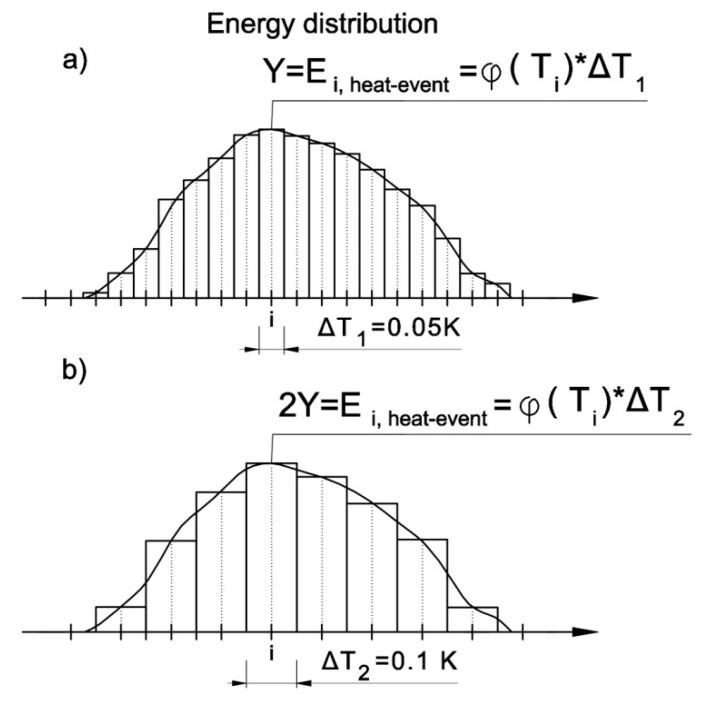
Influence of temperature interval widths on energy value, (**a**) 0.05 K wide interval, (**b**) 0.1 K wide interval.

Note that the assumed width of the ΔT intervals affects the value of energy, in particular the intervals of the energy distribution plot, and thus influences both the apparatus function that is assigned to them and the resolution of the calculations (Figure 6).

The following assumptions related to the apparatus function were made:

- Temperature functions have the same shape regardless of the temperature at which the thermal pulse is located (i.e., the only variable on the basis of which the apparatus function is selected is the value of the thermal pulse expressed in joules of energy).

- Apparatus functions that are obtained on the basis of ceramic sample tests may be used for testing other materials (thermal conductivity of the skeleton of the sample is negligible).

## 3. Algorithm Based on the Thermal Inertia of the Measurement System

Since the calorimetric curve depends on the value of the thermal pulse, which is unknown at the beginning of calculations, an original algorithm that involves approximations of the true distribution of thermal pulses and the corresponding apparatus functions was used.

**Figure 7:**
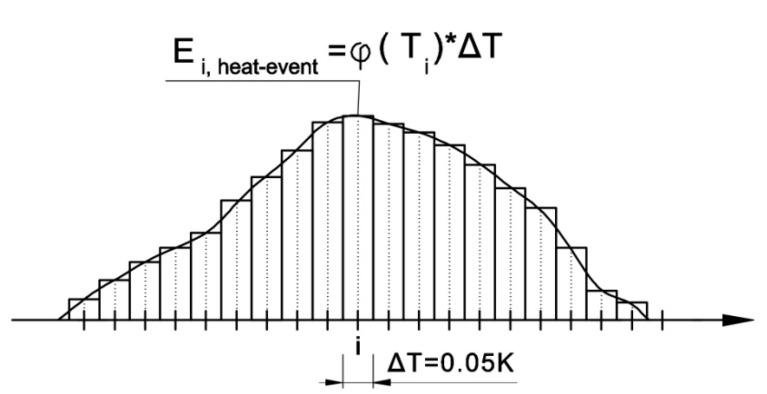
Schematic of the breakdown of the thermal capacity (*φ*(*T*)) signal into segments 0.05 K in wide with the amount of energy in each segment *E_i,heat-event_*.

The first step was to divide the recorded signal into segments 0.05 K in width and calculate the amount of energy in each segment (*E_i,heat-event_*) according to Figure 7. As a result, a series of single thermal pulses with (*E_i,heat-event_*) that were assigned to specific temperatures (initial energy distribution) was obtained.

(5) $$E_{i,heat-event}=\varphi \left(T_{i}\right)\Delta T$$

**Figure 8:**
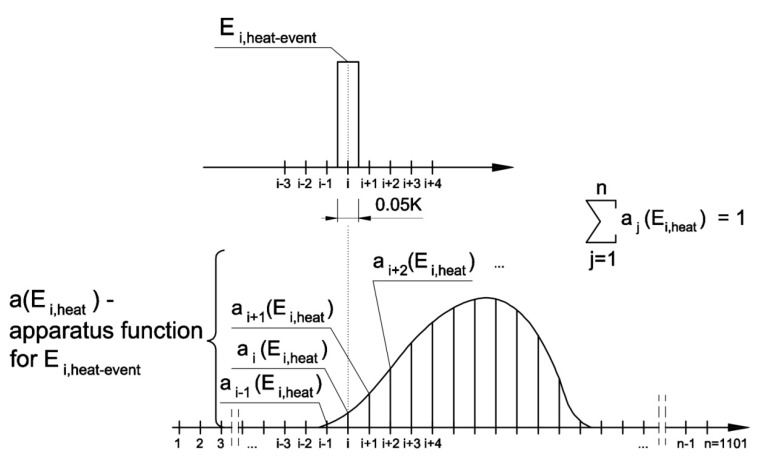
Apparatus function for the single thermal pulse E_i,heat-event_.

Apparatus functions *a*(*E_i,heat_*) were determined for each single pulse (Figure 8) according to Equation (4). Knowing that the selection of the apparatus function for a given pulse (*E_i,heat-event_*) depends on the values of neighboring pulses, the energy on the basis of which the apparatus function was determined is the sum of energies of the *i*-th thermal pulse, its preceding pulse, and the one that followed, according to Equation (6):

(6) $$E_{i,heat}=\overset{i+1}{\underset{j=i-1}{\sum }}E_{j,heat-event}$$

**Figure 9:**
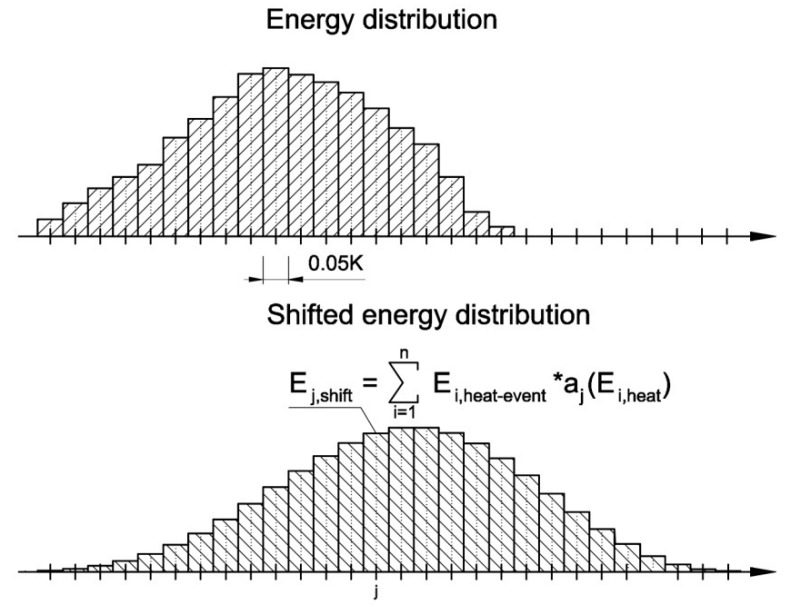
Shifted energy distribution calculated on the basis of thermal pulse values and the corresponding apparatus functions.

Then, on the basis of the initial energy distribution, the shifted energy distribution was calculated from Equation (7), as shown in Figure 9.

(7) $$E_{j,shift}=\overset{n}{\underset{i=1}{\sum }}E_{i,heat-event}a_{j}\left(E_{i,heat}\right)$$

**Figure 10:**
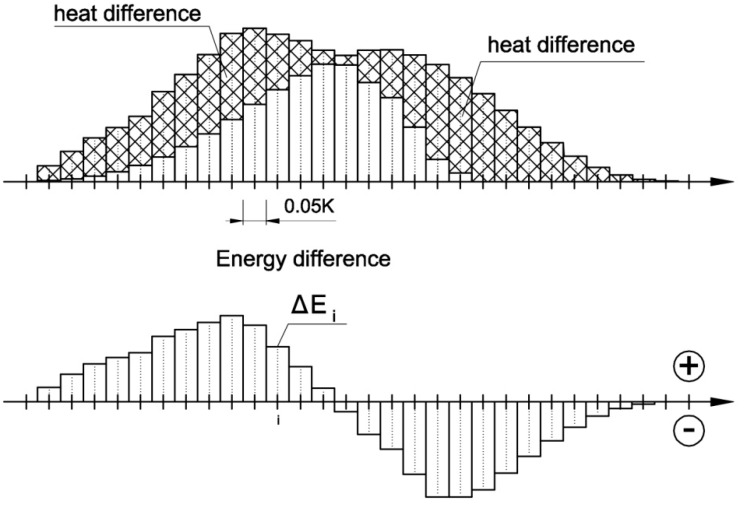
Energy difference between the energy distribution graph and the shifted energy distribution graph.

Finally, the differences between the initial and the shifted energy distributions were determined (Figure 10).

(8) $$\Delta E_{i}=E_{i,heat-event}-\;E_{i,shift}$$

**Figure 11:**
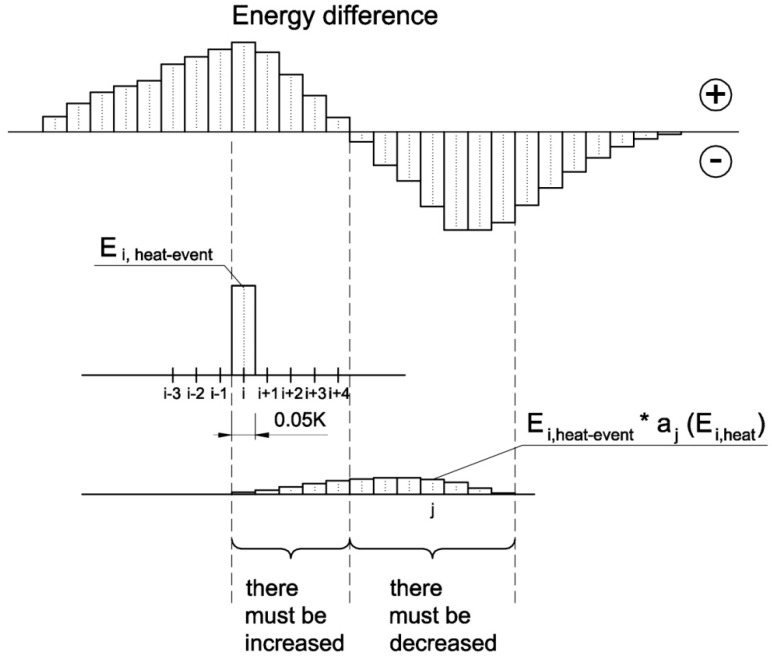
Schematic of heat pulse segments to be increased or decreased depending on the energy differences data.

With the energy differences and the known the shifted energy distribution, it was possible to, respectively, increase or decrease individual heat pulses according to Equation (9) and to obtain the first approximation of the energy distribution (Figure 11).

(9) $$E_{i,aprox}=E_{i,heat-event}+\;\overset{n}{\underset{j=1}{\sum }}\frac{\Delta E_{j}}{max\left(E_{j,heat-event},E_{j,shift}\right)}a_{j}\left(E_{i,heat}\right)w$$

where *w* = 0.1.

In subsequent cycles, *E_i,aprox_* was used to compute the shifted energy distribution by comparing it to the initial energy distribution.

With consecutive cycles, the values of *E_i,aprox_* became closer to the true values, and, in this way, the effect of the thermal inertia of the measurement system could be eliminated and the effect related to water melting at individual temperatures could be separated.

## 4. Calculations

Appendix A describes the procedure for the phase change energy calculation from a calorimetric signal.

When the spontaneous nucleation ends, the freezing process depends on the pore neck sizes [14]. Melting provides information about the pore internal sizes. The studies of Homeshaw et al. [23] and Kjeldsen et al. [18] showed that heating thermograms ensure a better assessment of the pore size distribution than cooling curves. In this paper, only some of the thermal effects that are associated with ice melting were used to investigate the pore space. To calculate the volume of pores in which ice melts at a given temperature, the latent heat *L*(*T*) (10) and ice density *ρ_ice_* (11) must be known. In this paper, the values given by Sun and Scherer [22] for ice melting in a cylindrical pore were used:

(10) $$L\left(T\right)\approx 333.8+1.797\cdot \left(T-T_{0}\right)$$

(11) $$\rho_{ice}\approx 0.9167-2.053\cdot 10^{-4}\cdot \left(T-T_{0}\right)-1.357\cdot 10^{-6}\cdot {\left(T-T_{0}\right)}^{2}$$

where *T* is pore liquid phase transition temperature and *K*, *T*_0_ is the freezing temperature of the bulk liquid.

The mass of ice melting at a given temperature interval *Δm*(*T*) can be obtained from the following equation:

(12) $$\Delta m\left(T\right)=\frac{E_{i,aprox}}{L\left(T_{i}\right)}$$

where *L*(*T_i_*) is the latent heat value at temperature *T_i_*.

The volume of the melting ice at a given temperature was calculated from the formula:

(13) $$\Delta V_{l}\left(T\right)=\frac{\Delta m\left(T\right)}{\rho_{ice}}$$

The equations above, when using the known values of both the exact phase transition temperature and the amount of energy, enabled the determination of the pore size distribution. In this paper, the equation proposed by Brun et al. [7] for ice melting (14) in a hypothetical cylindrical pore was used:

(14) $$r_{p}\;=-\frac{32.33}{T-T_{0}}+0.68\;\left(\mathrm{nm}\right)$$

Water close to pore walls does not freeze. The thickness of molecular layer that does not solidify was assumed to be equal *δ* = 0.8 nm. The volume of the pore was calculated from Equation (15):

(15) $$\Delta V_{l}\left(T\right)=\Delta V_{l}\left(T\right){\left(\frac{r_{p}}{r_{p}-\delta }\right)}^{n}$$

For cylindrical pores, *n* = 2.

## 5. Sample Preparation and Experiment

The application of the algorithm presented above was shown by using a mortar sample that was made with cement CEM II/B-V 32,5 [24] (w/c = 0.53). The fresh mortar mixture was degassed. A sample of fresh mortar was placed in a mold with dimensions of 15 × 15 × 15 cm. After removing the mold, the sample was stored in water for seven days and under dry conditions (temperature 20 ± 2 °C, relative humidity 50 ± 5%) for 80 days. Then, the DSC sample (ø13.5 × 70 mm) was obtained by using core drilling. Prior to performing the test, the specimen was dried at 105 °C and vacuum saturated with degassed distilled water. The measurement was performed by using a differential scanning calorimeter (model BT2.15CS SETARAM). The scanning program included cooling the sample from +20 to −80 °C, allowing it to stabilize at −80 °C for 30 minutes, and the reheating it to +20 °C. The calorimetric block was cooled with liquid nitrogen. The rate of cooling and heating was exactly 0.09 °C/min (5.4 °C/h). The sample was weighed and wrapped in Teflon foil before being placed in the calorimeter chamber to avoid evaporation. After completing the measurement, the samples was reweighed.

## 6. Results

**Figure 12:**
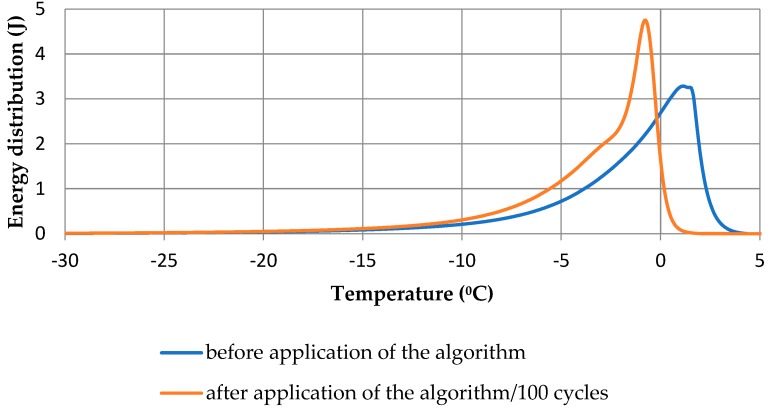
Distribution of phase transition energy of the CEMII/B-V cement mortar, w/c = 0.53.

Figure 12 shows the change in energy distribution when the algorithm (Section 3), which took the thermal inertia of the measuring system into account, was used. Even small energy shifts over the temperature range from −2 to 0 °C affected the radius to which a given amount of energy was assigned and hence, the calculated pore size distributions. Note that a portion of the energy was still assigned to temperatures greater than 0 °C.

Assigning the recorded signals to lower temperatures caused the corresponding amounts of energy to be divided by the lower value of the latent heat, and, thus, the calculations gave a higher total mass of ice. On basis of the initial energy distribution, the mass of ice equaled 1.08 g and increased to 1.09 g after the application of the algorithm.

**Figure 13:**
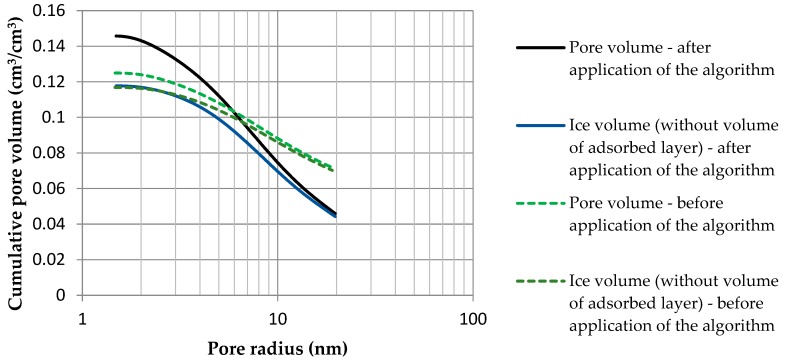
Pore size distribution that was obtained by thermoporometry from the phase transition energy distribution with the use of the modified algorithm.

The second factor that influenced the difference between the total volume of pores without accounting for thermal inertia of the measuring system and after applying the algorithm was the assumed volume of a film of non-freezable water, which was strongly adsorbed on pore wall surfaces. The relative portion of unfrozen water increased with the decrease in pore size (Figure 13).

**Figure 14:**
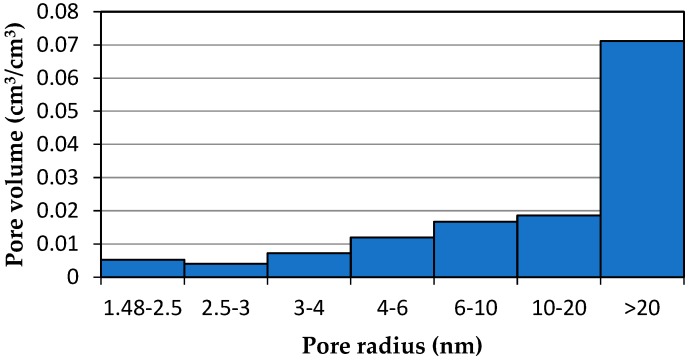
Pore size distribution that was obtained with thermoporometry on the basis of phase transition energy distribution, without taking thermal inertia into account.

**Figure 15:**
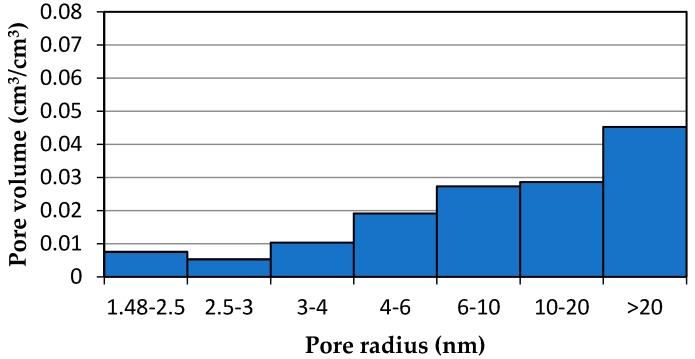
Pore size distribution that was obtained by thermoporometry from the phase transition energy distribution with the use of the modified algorithm. Running the proposed algorithm on the obtained phase transition energies was paramount to correctly determine the pore size distribution and, consequently, to predict the durability of materials. This knowledge provides an explanation of material properties, including the amount of capillary water in a unit of time, strength, and resistance to an aggressive environment.

Thus, the calculated pore volume increased proportionally to the increase in the amount of adsorbed water (Table 2). The example of the studied cement mortar showed that by taking the apparatus function into account, the decrease in the calculated volume of pores that were larger than 20 nm nearly doubled (Figure 14 and Figure 15).

## 7. Conclusions

If the process of the reasoning underlying the calculation procedure with the algorithm that accounts for the thermal inertia of the measurement system is correct, the size of pores and their distribution in cement mortar can be described with much greater precision.

In the case of the tested CEMII/B-V mortar (w/c = 0.53), 38.6% of the total ice–water energy of phase transition *E_sum_* was recorded over the temperature range above 0 °C. This is obviously a result that diverges from physical knowledge. When the energy distribution was used without taking the proposed modified algorithm into account, the volume of the relatively largest pores was greatly overestimated, while that of the smaller pores (*r_p_* < 20 nm) was underestimated, in some cases by 70%.

The proposed calculation formula can be used to analyze pore structure in other capillary and porous materials.

## Figures and Tables

**Table 1:** Total energy of phase transition.

Sample no.	E_sum_—Total Energy of Phase Transition	Designation
1	193.8 J	SAMPLE 1
2	586.5 J	SAMPLE 2
3	595 J	SAMPLE 3
4	1039 J	SAMPLE 4
5	1195 J	SAMPLE 5

**Table 2:** Pore volume.

Pore Radius (nm)	Pore Volume before Application of the Algorithm (cm^3^/cm^3^)	Pore Volume after Application of the Algorithm (cm^3^/cm^3^)
1.48–2.5	0.00525	0.00763
2.5–3	0.00403	0.00537
3–4	0.00718	0.01051
4–6	0.01195	0.01946
6–10	0.01667	0.02774
10–20	0.01859	0.02903
>20	0.07114	0.04597

**Figure A1:**
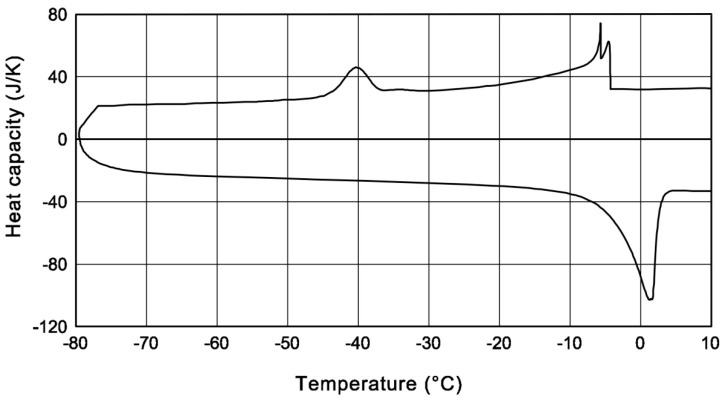
Difference in heat capacity *φ*(*T*) between the pan with the water saturated sample and the pan with the dry sample of the CEM II/B-V mortar, w/c = 0.53.

**Figure A2:**
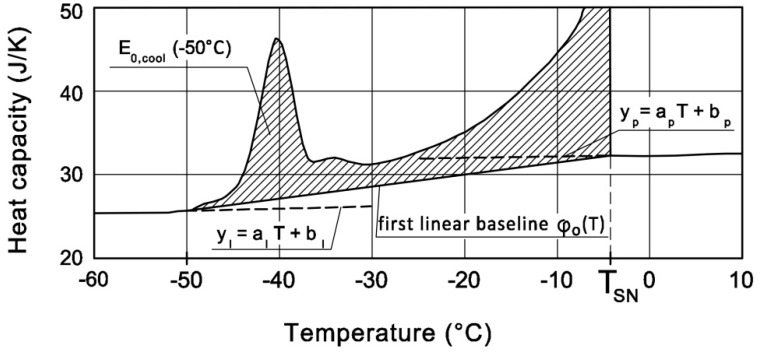
Heat capacity plot with marked lines y_p_, y_l_ and *φ*_0_(*T*).

**Figure A3:**
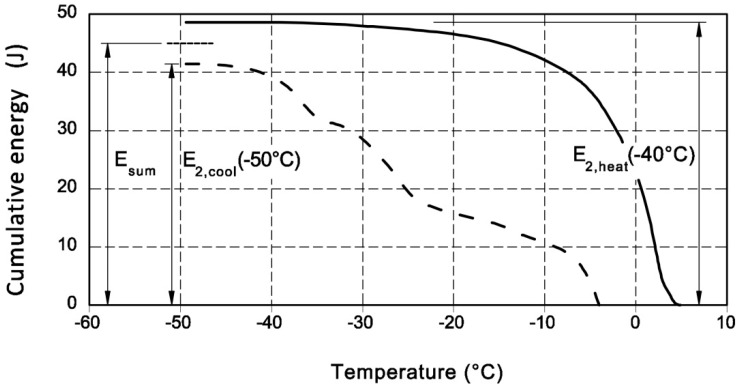
Schematic of determination of the total amount of phase transition energy.

## Appendix A

Figure A1 shows the heat capacity *φ*(*T*) diagram that was obtained as a result of the calorimetric measurements.

To separate part of the signal that was related only to the phase transition, a *φ*_2_(*T*) reference curve was constructed according to the procedure described below. Separately produced *φ*_2_(*T*) curves for cooling and heating were used to plot the accumulated energy of the water–ice phase transition, *E*_2,*cool,*Δ**_(*T*) (for cooling), and *E*_2,*heat,*Δ**_(*T*) (for heating).

The algorithm for the *φ*_2_(*T*) baseline:

In the first step, a reference straight line *φ*_0_(*T*) was determined. It linked the *φ*(*T*) values for the freezing start temperature *Tsn* and the temperature of −50 °C.

In the next step, the amounts of energy *E*_0,*cool*_(−50 °C) per the area between the *φ*_0_(*T*) and *φ*(*T*) for the temperature range from the spontaneous nucleation onset *T_SN_* and −50 °C were calculated from (A1).

(A1) $$E_{0,cool}\left(-50\;{}^{\circ}C\right)=\int_{T_{sn}}^{-50}\left(\varphi \left(T\right)-\varphi_{o}\left(T\right)\right)dT$$

Then, the coefficients of the straight lines y_p_ = a_p_T + b_p_ and y_l_ = *a*_l_T + *b*_l_ were found via the interpolation of the *φ*(*T*) curve for the section before the freezing onset and the section (−60 and −50 °C), respectively. These lines are shown in Figure A2. For the lines y_p_, y_l_ and energy *E*_0,*cool*_, the first reference curve *φ*_1_(*T*) had to be determined from the formulas:

(A2) $$\varphi_{1}\left(T\right)=a_{1}\left(T\right)\cdot T+b_{1}\left(T\right)$$

(A3) $$a_{1}\left(T\right)=\frac{\int_{T_{sn}}^{T}\left(\varphi \left(T\right)-\varphi_{o}\left(T\right)\right)dT}{E_{0,cool}\left(-50\;{}^{\circ}C\right)}a_{l}+\frac{E_{0,cool}-\int_{T_{sn}}^{T}\left(\varphi \left(T\right)-\varphi_{o}\left(T\right)\right)dT}{E_{0,cool}\left(-50\;{}^{\circ}C\right)}a_{p}$$

(A4) $$b_{1}\left(T\right)=\frac{\int_{T_{sn}}^{T}\left(\varphi \left(T\right)-\varphi_{o}\left(T\right)\right)dT}{E_{0,cool}\left(-50\;{}^{\circ}C\right)}b_{l}+\frac{E_{0,cool}-\int_{T_{sn}}^{T}\left(\varphi \left(T\right)-\varphi_{o}\left(T\right)\right)dT}{E_{0,cool}\left(-50\;{}^{\circ}C\right)}b_{p}$$

For the thus constructed *φ*_1_(*T*) curve, the amount of energy was calculated per the area between the *φ*_1_(*T*) curve and the heat capacity curve *φ*(*T*) for the temperature interval in the *T_SN_* and −50 °C range, according to Formula (A5).

(A5) $$E_{1,cool}\left(-50\;{}^{\circ}C\right)=\int_{T_{sn}}^{-50}\left(\varphi \left(T\right)-\varphi_{1}\left(T\right)\right)dT$$

Starting with the first step of the algorithm, the same procedure was repeated, but *φ*_1_(*T*), not *φ*_0_(*T*), was taken as the reference curve. The reference curve was determined again, and *φ*_2_(*T*) and the related amount of energy *E*_2,*cool*_(−50 °C) were obtained.

(A6) $$\varphi_{2}\left(T\right)=a_{2}\left(T\right)\cdot T+b_{2}\left(T\right)$$

(A7) $$a_{2}\left(T\right)=\frac{\int_{T_{sn}}^{T}\left(\varphi \left(T\right)-\varphi_{1}\left(T\right)\right)dT}{E_{1,cool}\left(-50\;{}^{\circ}C\right)}a_{l}+\frac{E_{0,cool}-\int_{T_{sn}}^{T}\left(\varphi \left(T\right)-\varphi_{1}\left(T\right)\right)dT}{E_{1,cool}\left(-50\;{}^{\circ}C\right)}a_{p}$$

(A8) $$b_{2}\left(T\right)=\frac{\int_{T_{sn}}^{T}\left(\varphi \left(T\right)-\varphi_{1}\left(T\right)\right)dT}{E_{1,cool}\left(-50\;{}^{\circ}C\right)}b_{l}+\frac{E_{0,cool}-\int_{T_{sn}}^{T}\left(\varphi \left(T\right)-\varphi_{1}\left(T\right)\right)dT}{E_{1,cool}\left(-50\;{}^{\circ}C\right)}b_{p}$$

(A9) $$E_{2,cool}\left(-50\;{}^{\circ}C\right)=\int_{T_{sn}}^{-50}\left(\varphi \left(T\right)-\varphi_{2}\left(T\right)\right)dT$$

The phase transition energy for heating from −80 to +20 °C (*E*_2,*heat*_) was determined in a similar way. The difference was that the temperature of the beginning of the melting effects was assumed to be −40 °C and not −50 °C. Two curves of accumulated phase transition energy *E*_2,*cool*_(*T*) and *E*_2,*heat*_(*T*) were thus obtained, as shown in Figure A3.

The accumulated phase transition energy *E*_2,*cool*_(*T*) and *E*_2,*heat*_(*T*) were averaged according to Formula (A10):

(A10) $$E_{sum}=\frac{E_{2,cool}\left(-50\;{}^{\circ}C\right)+E_{2,heat}\left(-40\;{}^{\circ}C\right)}{2}$$

The value of the calculated energy *E_sum_* was taken as the total amount of energy of the water phase transition during heating or cooling.

The plots of *E*_2,*cool*_(*T*) and *E*_2,*heat*_(*T*) needed to be corrected |*E_sum_* – *E*_2,*cool*_(−50 °C)| following the equations below:

(A11) $$E_{2,cool,\Delta }\left(T\right)=E_{2,cool}\left(T\right)\pm \left|E_{sum}+E_{2,cool}\left(-50\;{}^{\circ}C\right)\right|\cdot \frac{\int_{T_{sn}}^{T}\left(\varphi \left(T\right)-\varphi_{2}\left(T\right)\right)dT}{E_{2,cool}\left(-50\;{}^{\circ}C\right)}$$

(A12) $$E_{2,heat,\Delta }\left(T\right)=E_{2,heat}\left(T\right)\pm \left|E_{sum}+E_{2,cool}\left(-50\;{}^{\circ}C\right)\right|\cdot \frac{\int_{-40}^{T}\left(\varphi \left(T\right)-\varphi_{2}\left(T\right)\right)dT}{E_{2,heat}\left(-40\;{}^{\circ}C\right)}$$

The plot of *E*_2,*heat,Δ*_(*T*) was the basis for calculating the energy of the melting ice over individual temperature intervals (0.05 K wide), and, in this way, the distribution of phase transition energy before applying the algorithm (Figure 12) was obtained.
